# Antibacterial and Antifungal Potential of some Arid Zone Plants

**DOI:** 10.4103/0250-474X.73939

**Published:** 2010

**Authors:** S. C. Jain, B. Pancholi, R. Singh, R. Jain

**Affiliations:** Medicinal Plants and Biotechnology Laboratory, Department of Botany, University of Rajasthan, Jaipur-302 004, India; 1 Department of Chemistry, University of Rajasthan, Jaipur-302 004, India

**Keywords:** Agar well diffusion, arid zone plants, minimum inhibitory concentration

## Abstract

Sequential extracts of some medicinally important arid zone plants of Rajasthan, viz. *Lepidagathis trinervis* Nees., *Polycarpea corymbosa* Lam. and *Sericostoma pauciflorum* Stocks. ex Wight. were tested against six bacterial (Gram +ve and Gram –ve) and five fungal strains using agar well diffusion method. Ethyl acetate extract of *L. trinervis* showed maximum activity against *Bacillus subtilis, Enterobactor aerogenes, Pseudomonas aeruginosa, Aspergillus flavus* and *Trichophyton rubrum* (inhibition zone 16.00±0.81, 13.33±0.66, 14.33±1.85, 14.30±0.34 and 23.00±0.00 mm) at varied minimum inhibitory concentrations of 82, 20, 41, 41 and 20 μg/ml, respectively.

*Lepidagathis trinervis* Nees (Acanthaceae) is a prostrate to sub-erect, up to 30 cm tall undershrub and its ashes were used to cure eczema[1]. *L. trinervis* shows anticancer activity against L_1210_ lymphoid leukemia and hypotensive effect[2]. *Polycarpea corymbosa* Lam. traditionally used in venomous bites from reptile, jaundice and on inflammatory swellings[3,4]. Some sterols such as α-1 barrigenol, camelliagenin A and stigmasterol have been isolated[5]. *Sericostoma pauciflorum* Stocks ex Wight (Boraginaceae) is a short straggling undershrub growing widely throughout sea coast of Saurashtra and Maharashtra. It is generally used in dehydration and acidity. Phytochemically, fernane, hopane and other type of triterpenoids were isolated from the plant[6–8].

Whole plants of each were collected from the fields locally during the months of October to January, 2007-08. The botanical identity was confirmed by Herbarium, Department of Botany, University of Rajasthan, Jaipur. Voucher specimens of the plants have been deposited at the Herbarium and Laboratory for further reference. Each of 100 g air-dried, powdered plant materials was Soxhlet extracted separately for 72 h in petroleum ether, dichloromethane, ethyl acetate, methanol and water in increasing order of polarity. The different extracts were concentrated and dried using vacuum evaporator to give solid residue and were stored at 4^0^, until use.

For antimicrobial screening, Gram +ve bacteria (*Bacillus subtilis* MTCC 441; *Staphylococcus aureus* MTCC 740) and Gram -ve bacteria (*Escherichia coli* MTCC 443; *Pseudomonas aeruginosa* MTCC 741; *Enterobacter aerogens* MTCC 111 and *Raoultella planticola* MTCC 530), obtained from IMTECH, Chandigarh, were used. The bacterial strains were maintained on nutrient agar medium. Further, fungi namely *Aspergillus niger* (ATCC 322), *A. flavus* (ATCC 16870), *Candida albicans* (ATCC 4718), *Trichophyton rubrum* (ATCC 2327) and *Penicillium crysogenum* (ATCC 5476) obtained from IARI, New Delhi, were used. These fungal strains were maintained on Sabouraud dextrose agar (SDA) medium.

Antimicrobial screening was performed by agar well diffusion method[9] using Müller-Hinton medium for antibacterial and SDA medium for antifungal activity. In the culture plates, wells were prepared with the help of sterile cork borer (6 mm in diameter) and 4 mg extract were delivered per well. Plates were incubated at 37^0^for bacteria and 25^0^in case of fungi for 24 h under aerobic conditions. The diameter of the inhibition zone (IZ) around each hole was measured by inhibition zone recorder (HiMedia) in triplicate and statistically analyzed.

Agar well diffusion method[9] was used for the determination of MIC of crude plant extracts. Serial dilutions of the extracts ranging 2000 μg to 20 μg were prepared and administered in previously inoculated plates. Gentamycin (10 μg/ml) in case of bacteria and ketoconozole (100 units/ml) in case of fungi were used as standard antibiotics.

Among Gram + ve bacteria, maximum activity was exhibited by *L. trinervis* ethyl acetate extracts against *B. subtilis* (IZ 16.00±0.81 mm, MIC 82 μg/ml; Table 1) and *P. corymbosa* petroleum ether extract against *S. aureus* (IZ 13.66±0.32 mm, MIC 62.5 μg/ml).

**TABLE 1:** ANTIBACTERIAL ACTIVITY OF SELECTED ARID ZONE PLANTS

Extracts		*B. subtilis*	*E. aerogenes*	*E. coli*	*P. aeruginosa*	*R. planticola*	*S. aureus*
*L. trinervis*							
Petroleum ether	IZ	10.00±0.00	11.66±066	10.00±0.33	11.66±1.02	10.33±0.32	10.00±0.00
	MIC	1000	1000	-	1000	2000	-
Dichloromethane	IZ	12.50±0.40	12.00±0.00	10.00±0.33	13.66±0.88	10.33±0.32	9.33±0.66
	MIC	125	250	1000	125	250	500
Ethylacetate	IZ	16.00±0.81	13.33±0.66	12.00±0.00	14.33±1.85	11.33±0.32	10.33±1.19
	MIC	82	20	500	41	500	500
Methanol	IZ	13.50±0.40	11.30±1.33	10.00±0.33	19.33±2.02	11.33±0.32	12.60±0.66
	MIC	82	-	2000	41	62.5	125
*P. corymbosa*							
Petroleum ether	IZ	13.00±1.00	10.00±0.57	12.66±0.67	10.00±0.57		13.66±0.32
	MIC	125	500	500	500	125	62.5
Dichloromethane	IZ	10.00±0.00	9.33±0.40	10.33±0.40	-	11.66±0.66	11.33±0.32
	MIC	1000	500	1000	-	250	500
Ethyl acetate	IZ	10.66±0.30	12.33±0.67	10.23±0.78	12.66±0.33	11.00±0.00	12.67±0.66
	MIC	250	500	125	250	500	500
Methanol	IZ	11.66±0.30	15.00±0.57	14.00±0.00	13.66±0.32	9.00±0.0	10.66±0.66
	MIC	1000	250	500	125	500	1000
Aqueous	IZ	9.66±0.30	13.33±0.33	8.00±0.00	19.00±0.00	10.00±0.00	10.00±0.66
	MIC	125	500	100	2000	100	1000
*S. pauciflorum*							
Petroleum ether	IZ	10.0±0.00	10.00±0.00	11.66±0.66	11.66±0.74	9.66±0.37	10.33±0.32
	MIC	-	500	500	-	1000	250
Dichloromethane	IZ	12.33±0.32	10.66±0.32	11.33±0.66	12.66±0.66	9.66±0.37	10.66±0.66
	MIC	330	500	-	82	250	250
Ethyl acetate	IZ	13.00±0.00	15.33±0.33	14.66±0.66	13.66±0.66	11.66±0.66	13.66±1.17
	MIC	41	82	330	82	62.5	500
Methanol	IZ	14.00±0.57	12.00±1.00	12.33±0.33	10.33±0.33	10.66±0.37	10.33±0.37
	MIC	-	250	1000	500	125	500
Aqueous	IZ	15.00±0.57	10.33±0.32	10.33±0.32	15.66±0.66	11.00±0.57	11.00±0.57
	MIC	125	-	-	125	2000	1000

All values are the mean±standard deviation or standard error of three determinations; IZ= Inhibition zone in mm; MIC= Minimum inhibitory concentration in µg/ml.

In case of Gram -ve bacteria, *L. trinervis* ethyl acetate extract showed maximum activity against *E. aerogens* (Inhibition Zone 13.33±0.66 mm, MIC 20 μg/ml) followed by ethyl acetate extract of *S. pauciflorum* (IZ 15.33±0.33 mm, MIC 82 μg/ml). Ethyl acetate and methanol extracts of *L. trinervis* exhibited maximum inhibition against *P. aeruginosa* (IZ 14.33±1.85 mm and 19.33±2.02 mm respectively, MIC 41 μg/ml in both cases). Methanol extract of *L. trinervis* (IZ 15.33±0.33 mm) and ethyl acetate extract of *S. pauciflorum* (IZ 11.66±0.66 mm) with MIC of 62.5 μg/ml demonstrated maximum inhibition against *R. planticola*.

Likewise maximum antifungal activity against *A. flavus* was demonstrated by dichloromethane and ethyl acetate extract of *L. trinervis* (IZ 16.00±0.00 mm, 14.30±0.33 mm with MIC 41 μg/ml; Table 2). Petroleum ether extract of *S. pauciflorum* showed significant inhibitory effect against *A. niger* (IZ 11.66±0.33 mm, with MIC 62.5 μg/ml). Appreciable activity against *T. rubrum* was exhibited by dichloromethane and ethyl acetate extracts of *L. trinervis* (IZ 20.00±0.00 mm and 23.00±0.00 mm, respectively with MIC 20 μg/ml in both cases).

**TABLE 2:** ANTIFUNGAL ACTIVITY OF SELECTED ARID ZONE PLANTS

Extracts	**	*A. flavus*	*A. niger*	*C. albicans*	*P. chrysogenum*	*T. rubrum*
*L. trinervis*						
Petroleum ether	IZ	14.30±0.33	11.00±0.00	10.00±0.00	12.00±0.00	17.50±0.40
	MIC	250	250	250	125	41
Dichloromethane	IZ	16.00±0.00	11.00±0.57	12.00±0.00	11.33±0.32	20.00±0.00
	MIC	41	1000	250	500	20
Ethyl acetate	IZ	14.30±0.33	13.00±0.81	11.66±0.34	11.00±0.00	23.00±0.00
	MIC	41	1000	250	500	20
Methanol	IZ	17.60±1.20	14.33±0.37	13.00±0.00	-	17.00±2.44
	MIC	1000	125	500	-	2000
*P. corymbosa*						
Petroleum ether	IZ	10.45±0.67	-	10.00±0.00	10.56±0.34	10.00±0.57
	MIC	1000	-	500	125	500
Dichloromethane	IZ	9.33±0.32	10.33±0.32	11.00±1.00	13.00±1.00	10.66±0.40
	MIC	250	500	125	500	250
Ethyl acetate	IZ	10.00±0.00	14.78±0.66	15.66±0.66	8.66±0.66	11.00±0.57
	MIC	500	500	250	125	1000
Methanol	IZ	10.30±0.32	17.00±0.00	10.00±0.00	10.00±0.57	10.66±0.67
	MIC	1000	500	250	250	250
Aqueous	IZ	13.33±0.40	14.66±0.30	15.00±1.00	10.66±0.67	10.00±0.57
	MIC	250	1000	125	125	125
*S. pauciflorum*						
Petroleum ether	IZ	12.66±0.66	11.66±0.33	12.66±0.66	11.66±0.32	10.00±0.00
	MIC	500	62.5	500	250	500
Dichloromethane	IZ	14.00±0.00	10.00±0.00	16.60±0.33	13.66±1.33	11.00±0.57
	MIC	1000	1000	62.5	82	62.5
Ethyl acetate	IZ	12.66±1.33	10.00±0.00	11.00±0.00	13.66±1.33	10.33±0.88
	MIC	500	250	125	41	125
Methanol	IZ	10.00±1.00	10.00±0.00	11.66±0.32	14.33±0.66	8.00±0.00
	MIC	250	250	500	500	500
Aqueous	IZ	10.00±0.00	12.66±0.74	11.66±0.32	13.33±0.32	9.33±0.32
	MIC	500	250	500	500	500

All values are the mean±standard deviation or standard error of three determinations; IZ= Inhibition zone in mm; MIC= Minimum inhibitory concentration in µg/ml.

From these results, it is evident that the selected plants demonstrated potential antibacterial and antifungal activities. In literature, *L. trinervis*, has been known as a bitter tonic and used to cure eczema[1] while *P. corymbosa* also used in suppression of inflammatory swellings[3,4], as reported elsewhere in Ayurvedic literature, have been further established.
